# A new species of *Pristimantis* from eastern Brazilian Amazonia (Anura, Craugastoridae)

**DOI:** 10.3897/zookeys.687.13221

**Published:** 2017-08-02

**Authors:** Elciomar Araújo De Oliveira, Luis Reginaldo Rodrigues, Igor Luis Kaefer, Karll Cavalcante Pinto, Emil José Hernández-Ruz

**Affiliations:** 1 Programa de Pós-Graduação em Biodiversidade e Biotecnologia da Rede BIONORTE, Universidade Federal do Amazonas, Av. Gen. Rodrigo Octávio Jordão Ramos, 3000, CEP 69077-000, Manaus, Amazonas, Brazil; 2 Programa de Pós-Graduação em Biociências, Universidade Federal do Oeste do Pará, Rua Vera Paz, s/n (Unidade Tapajós), CEP 68035-110, Santarém, Pará, Brazil; 3 Universidade Federal do Amazonas. Av. Gen. Rodrigo Octávio Jordão Ramos, 3000, CEP 69077-000, Manaus, Amazonas, Brazil; 4 Biota Projetos e Consultoria Ambiental LTDA, Rua 86-C, 64, CEP 74083-360, Setor Sul, Goiânia, Goiás, Brazil; 5 Programa de Pós-Graduação em Biodiversidade e Conservação, Faculdade de Ciências Biológicas, Campus Universitário de Altamira, Universidade Federal do Pará, Rua Coronel José Porfírio, 2515, CEP 68372-040, Altamira, Pará, Brazil

**Keywords:** mitochondrial DNA, *Pristimantis latro* sp. n., systematics, Terrarana

## Abstract

In this study a new species of *Pristimantis* (Anura: Craugastoridae) of the *P. conspicillatus* species group is described. *Pristimantis latro* **sp. n.** is known only from the municipalities of Altamira, Anapu, Brasil Novo, Medicilândia, Uruará and Aveiro (Flona Tapajós, right bank of Tapajós river), in Pará state, Brazil. Morphologically, the new species distinguishes from known congeners in the group mainly by the presence of dorsal tubercles and absence of discoidal folds, smooth belly skin, as well as the presence of supernumerary tubercles on hands. The call of the new species consists of seven ascending notes, the first of which has a dominant frequency of 2635 Hz and the last 3272 Hz. Molecular analysis of the 16S mtDNA indicates a genetic distance of 8% to *P. chiastonotus*, its closet relative, and between 9% and 11% to populations of *P. fenestratus*.

## Introduction

The genus *Pristimantis* Jiménez de la Espada, 1870, currently contains 506 described species (Frost 2017). *Pristimantis* is the largest genus among all vertebrates (Fouquet et al. 2013) and its remarkable diversity could probably be explained by the evolution of direct development, allowing individuals not to rely on water bodies for reproduction and thus making them fit for niches unoccupied by other amphibians (Terán-Valdez and Guayasamin 2010). Another important feature of the genus is its highly variable body size, varying from 14.5 mm (*P. andinognomus* Lehr & Coloma, 2008) up to 73.0 mm (*P. lymani* Barbour & Noble, 1920) (Hedges et al. 2008), a factor also likely to have increased the exploitation of various niches. The *P. conspicillatus* group (Lynch and Duellman 1997) contains 33 species (Padial et al. 2014) distributed in east Honduras through Central America, Colombia and Ecuador to Peru, Bolivia, northern Argentina, Atlantic and Amazonian Forests in Brazil and the Guianas, Trinidad and Tobago, and Grenada, Lesser Antilles (Frost 2017).

**Figure 1.**
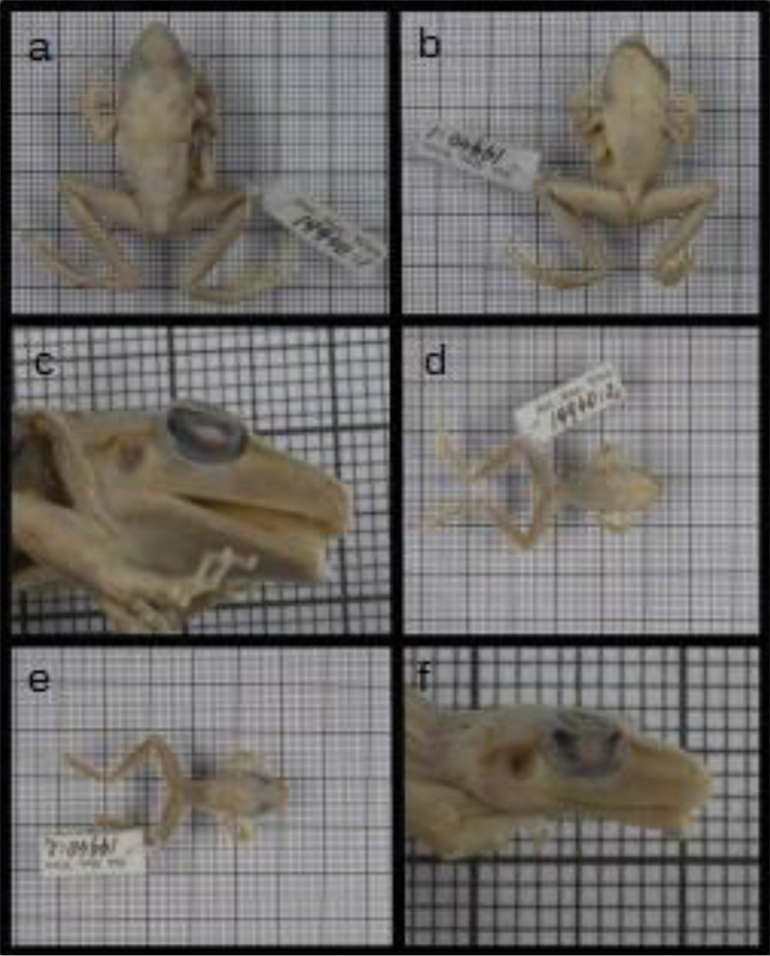
Lectotype of *Pristimantis fenestratus* from Rio Mamoré, Rondônia, Brazil. **A** dorsal view **B** ventral view **C** lateral view of the head. Paralectotype from the municipality of Borba, Amazonas, Brazil **D** dorsal view **E** ventral view **F** lateral view of the head.

The species *P. fenestratus* (Steindachner, 1864) belongs to the *P. conspicillatus* group and has a wide distribution in the Amazon region (Lima et al. 2006; Bernarde and Macedo 2008; França and Venâncio 2010; Ávila-Pires et al. 2010). The taxonomy of *P. fenestratus* is problematic because many morphologically different populations have been wrongly included under that name (Duellman and Lehr 2009; Siqueira et al. 2009). This can be, at least partly, attributed to inconsistencies regarding the type locality, which was suggested to be the upper Madeira River region by De la Riva et al. (2000) and the lower Madeira River by Siqueira et al. (2009). According to Häupl and Tiedemann (1978) and Häupl et al. (1994), *P. fenestratus* has two syntypes: NHMW 19940.1 (Río Mamoré) and 19940.2 (Borba). Reichle (1999), visiting the Naturhistorisches Museum Wien (NMW), designated the syntype NMW 19940:1 (Figure 1a, b, c) from the Mamoré River, Rondônia State, Brazil, as lectotype of *Pristimantis fenestratus* and the syntype NMW 19940:2 (Figure 1d, e, f) from the municipality of Borba, Amazonas State, Brazil, as a paralectotype. De la Riva et al. (2000) and Padial and De la Riva (2009) considered populations of *P. fenestratus* collected in the ‘Rio Mamoré’ (Bolivian Amazon) and adjacent Andes slopes as conspecific with *P. fenestratus* from the locality of the lectotype.

Here, using morphological, molecular, and bioacoustics data, we describe a new species of *Pristimantis* of the *P. conspicillatus* group that is morphologically similar to *P. fenestratus* and *P. koehleri*.

## Materials and methods

### Morphological analysis

Thirteen individuals of the Coleção Zoológica de Anfíbios e Répteis from the Instituto Nacional de Pesquisas da Amazônia (INPA-H), six of the Coleção Herpetológica of the Museu Paraense Emílio Goeldi (MPEG) and 69 (twenty-two belonging to type series) of the Coleção de Répteis e Anfíbios of the Universidade Federal do Pará/Campus de Altamira (Appendix 1) were examined, totaling 88 individuals identified as Pristimantis aff. fenestratus. Direct comparisons of character states were performed with nine specimens of *P. fenestratus* from the municipality of Borba, Amazonas state, Brazil, deposited in the Coleção Zoológica de Anfíbios e Répteis from the Instituto Nacional de Pesquisas da Amazônia (INPA-H). The gathered information was then compared with descriptions from the literature (Duellman and Lehr 2009; Padial and De La Riva 2009).

The morphological characters were described according to the suggested nomenclature summarized in Kok and Kalamandeen (2008), Padial and De la Riva (2009) and Duellman and Lehr (2009): 1) belly skin texture (smooth or granular); 2) dorsal tubercles (present or absent); 3) fringes on fingers (present or absent); 4) dorsolateral folds (present or absent); 5) fringes on foot (prominent, weak or absent); 6) basal toe webbing (present or absent); 7) tarsal fold (prominent, weak or absent); 8) color pattern of throat, chest and belly (heavily spotted, weakly spotted, immaculate); 9) supernumerary palmar tubercles (present or absent); 10) external palmar tubercle (entire, bifid, or semi-bifid).

Measurements were taken with a digital caliper to the nearest 0.01 mm and rounded to the nearest 0.1 mm as in Kok and Kalamandeen (2008), Padial and De la Riva (2009) and Duellman and Lehr (2009). The measurements obtained are as follows:

**SVL** Snout-Vent Length (from tip of snout to posterior margin of vent)

**HL** Head Length (from posterior margin of lower jaw to tip of snout)

**HW** Head Width (measured at level of rictus)

**SL** Snout Length (from anterior corner of eye to tip of snout)

**DEN** Distance from Eye to Nostril (from anterior corner of eye to posterior margin of naris)

**ID** Internarial Distance (taken between the median margins of the nares)

**EL** Eye Length (measured horizontally)

**IoD** Interorbital Distance (taken between the inner margins of the orbits)

**EW** Eyelid Width (the largest transverse width of the upper eyelid)

**TL** Tympanum Length (the largest length of the tympanum from the anterior margin to the posterior margin of the tympanum)

**AL** Arm Length (from the tip of the elbow to the proximal edge of the palmar tubercle)

**HaL** Hand Length (from the proximal edge of the palmar tubercle to the tip of Finger III),

**ThL** Thigh Length (from vent to knee)

**TiL** Tibia Length (from outer edge of flexed knee to heel)

**TaL** Tarsus Length (from the heel to the proximal edge of the inner metatarsal tubercle)

**FL** Foot Length (from proximal border of inner metatarsal tubercle to tip of fourth toe)

**LL** Leg Length (from the knee joint to the tip of Toe IV).

Sex and maturity were determined by direct examination of gonads through a lateral incision in the abdomen. In addition, we checked for secondary sexual characters in adult individuals, such as the presence or absence of vocal slits, vocal sac, and nuptial pads in males.

### Bioacoustic analysis

Recordings of advertisement calls were obtained from six males of the new species: one male was recorded on February 10, 2016 between 17:30 h and 18:00 h, from a distance of 2 m in Brazil Novo, Pará, at a temperature of 28 ºC. Five additional males were recorded on February 17, 2017 between 18:30 h and 20:00 h from a distance of 2 m in Altamira, Pará, at a temperature of 28 ºC. The vocalizations of *Pristimantis koehleri*, *P. fenestratus* and *P. samaipatae* (Köhler & Jungfer, 1995) available in the literature were used for comparisons with the new species. These are commonly used in descriptive bioacoustic studies (Padial and De La Riva 2009; Maciel et al. 2012). Data on the advertisement call of *P. chiastonotus* was obtained from the study of Lynch and Hoogmoed (1977).

The calls were analysed at a sampling rate of 44100 Hz using Audacity 2.0.3 software for Windows (Free Software Foudation Inc. 1991). Frequency information was obtained through Fast Fourier Transformations (FFT; width of 1024 points). Spectrograms and oscillograms were generated using Praat 5.3.43 for Windows (Boersma and Weenink 2006), following Yu and Zheng (2009), Zhou et al. (2014), and Preininger et al. (2016). The following variables were measured according to Padial and De la Riva (2009): call length (ms), number of notes per call, length of the note (ms), presence of pulses, fundamental frequency (frequency band to which the first sound is visualized through a spectral slice output, in Hz) and dominant frequency (measured from a spectral slice taken from the highest amplitude portion of the note, in Hz), in Praat 5.3.43 software.

### Molecular analysis

Total genomic DNA was extracted from 46 specimens (Table 1) using the CTAB 2% protocol (Doyle and Doyle 1987). A fragment of 490 base pairs (bp) of the 16S mtDNA was amplified by PCR using primers 16Saf and 16Sbr (Palumbi 1991). Amplification was performed under the following conditions: 60s at 92 °C followed by 35 cycles of 92 °C (60 sec), 50 °C (50 sec) and 72 °C (90 sec). The final volume of the PCR reaction was 12 μL and contained 4.7 μL of ddH_2_O, 1.5 μL of 25 mM MgCl_2_, 1.25 μL of 10 mM dNTPs (2.5 mM each dNTP), 1.25 μL of tampon 10x (75 mM Tris HCl, 50 mM KCl, 20 mM (NH_4_)_2_SO_4_), 1 μL of each primer (2 μM), 0.3 μL of 1 U Taq DNA Polymerase and 1 μL of DNA (30–50 ng/μL).

**Table 1.** List of specimens used for molecular analysis.

Species	Localities	GenBank	Nº in collection	Status of specimens	Source
*Pristimantis latro* sp. n.	Anapu, PA - Brazil	KX242519	LZATM 467	Holotype	this study
*Pristimantis* sp. n.	Anapu, PA - Brazil	KX925980	LZATM 743	Paratype	this study
*Pristimantis* sp. n.	Anapu, PA - Brazil	KX925981	LZATM 739	Paratype	this study
*Pristimantis* sp. n.	Anapu, PA - Brazil	KX925983	LZATM 744	Paratype	this study
*Pristimantis* sp. n.	Sen. José Porfírio, PA - Brazil	KX925984	LZATM 742	Paratype	this study
*Pristimantis* sp. n.	Sen. José Porfírio, PA - Brazil	KX925985	LZATM 748	Paratype	this study
*Pristimantis* sp. n.	Sen. José Porfírio, PA - Brazil	KX925986	LZATM 751	Paratype	this study
*Pristimantis* sp. n.	Altamira, PA - Brazil	KX925987	LZATM 386	Paratype	this study
*Pristimantis* sp. n.	Altamira, PA - Brazil	KX925988	BIOTA 1218	Paratype	this study
*Pristimantis* sp. n.	Altamira, PA - Brazil	KX925989	BIOTA 1111	Paratype	this study
*Pristimantis* sp. n.	Altamira, PA - Brazil	KX242523	BIOTA1214	Paratype	this study
*Pristimantis* sp. n.	Altamira, PA - Brazil	KX242523	LZATM 213	Paratype	this study
*Pristimantis* sp. n.	Altamira, PA - Brazil	KX242522	LZATM 277	Paratype	this study
*Pristimantis* sp. n.	Altamira, PA - Brazil	KX925990	LZATM 279	Paratype	this study
*Pristimantis* sp. n.	Altamira, PA - Brazil	KX925991	LZATM 281	Paratype	this study
*Pristimantis* sp. n.	Medicilândia, PA - Brazil	KX925992	LZATM 230	Paratype	this study
*Pristimantis* sp. n.	Medicilândia, PA - Brazil	KX925993	LZATM 243	Paratype	this study
*Pristimantis* sp. n.	Medicilândia, PA - Brazil	KX925994	LZATM 255	Paratype	this study
*Pristimantis* sp. n.	Flona Tapajós, PA - Brazil	KX242525	SISTAP 1145	Paratype	this study
*Pristimantis* sp. n.	Flona Tapajós, PA - Brazil	KX925995	SISTAP 1168	Paratype	this study
*Pristimantis* sp. n.	Flona Tapajós, PA - Brazil	KX925996	SISTAP 1235	Paratype	this study
*Pristimantis* sp. n.	Flona Tapajós, PA - Brazil	KX242524	SISTAP 1239	Paratype	this study
*Pristimantis* sp. n.	Flona Tapajós, PA - Brazil	KX925997	SISTAP 1240	Paratype	this study
*Pristimantis* sp. n.	Flona Tapajós, PA - Brazil	KX925998	SISTAP 1244	Paratype	this study
*Pristimantis* sp. n.	Flona Tapajós, PA - Brazil	KX925999	SISTAP 1246	Paratype	this study
*Pristimantis* sp. n.	Flona Tapajós, PA - Brazil	KX926000	SISTAP 1253	Paratype	this study
*Pristimantis* sp. n.	Flona Tapajós, PA - Brazil	KX926001	SISTAP 1256	Paratype	this study
*Pristimantis* sp. n.	Flona Tapajós, PA - Brazil	KX926002	SISTAP 1257	Paratype	this study
*Pristimantis* sp. n.	Flona Tapajós, PA - Brazil	KX926003	SISTAP 1259	Paratype	this study
*Pristimantis* sp. n.	Flona Tapajós, PA - Brazil	KX926004	SISTAP 1260	Paratype	this study
*Pristimantis* sp. n.	Flona Tapajós, PA - Brazil	KX926005	SISTAP 1261	Paratype	this study
*Pristimantis* sp. n.	Flona Tapajós, PA - Brazil	KX926006	SISTAP 1275	Paratype	this study
*Pristimantis* sp. n.	Flona Tapajós, PA - Brazil	KX926007	MPEG 095	Paratype	this study
*Pristimantis* sp. n.	Flona Tapajós, PA - Brazil	KX926008	MPEG 109	Paratype	this study
*Pristimantis* sp. n.	Flona Tapajós, PA – Brazil	KX926009	MPEG 160	Paratype	this study
*Pristimantis* sp. n.	Flona Tapajós, PA - Brazil	KX926010	MPEG 165	Paratype	this study
*Pristimantis* sp. n.	Flona Tapajós, PA - Brazil	KX926011	MPEG 177	Paratype	this study
*P. fenestratus*	Borba 2, AM – Brazil	KX242528	INPA-H 34565	Voucher	this study
*P. fenestratus*	Borba 2, AM – Brazil	KX926012	INPA-H 34580	Voucher	this study
*P. fenestratus*	Borba 2, AM – Brazil	KX926013	INPA-H 34579	Voucher	this study
*P. fenestratus*	Borba 1, AM – Brazil	KX242530	INPA-H 34571	Voucher	this study
*P. fenestratus*	Borba 1, AM – Brazil	KX926014	INPA-H 34577	Voucher	this study
*P. fenestratus*	Borba 1, AM – Brazil	KX926015	INPA-H 34562	Voucher	this study
*P. fenestratus*	Borba 1, AM – Brazil	KX242529	INPA-H 34573	Voucher	this study
*P. fenestratus*	Borba 1, AM – Brazil	KX926016	INPA-H 34578	Voucher	this study
*P. fenestratus*	Borba 1, AM – Brazil	KX926017	INPA-H 34575	Voucher	this study
*P. koehleri*	Bolívia, Santa Cruz	EU192278	MNCN 42990	Paratopotype	Padial and De La Riva 2009
*P. koehleri*	Bolivia, Santa Cruz	EU192279	MNCN 6627	Paratopotype	Padial and De La Riva 2009
*P. koehleri*	Bolivia, Santa Cruz	EU192280	MNCN 42983	Paratype	Padial and De La Riva 2009
*P. koehleri*	Bolivia, Santa Cruz	EU192281	MNCN 43013	Paratype	Padial and De La Riva 2009
*P. koehleri*	Bolivia, Santa Cruz	EU192282	MNCN 42986	Paratype	Padial and De La Riva 2009
*P. fenestratus*	Bolivia, La Paz: Chalalan	EU192273	MNKA 6629	Voucher	Padial and De La Riva 2009
*P. fenestratus*	Bolivia, La Paz	EU192274	MNKA 6630	Voucher	Padial and De La Riva 2009
*P. fenestratus*	Bolivia, Cochabamba	EU192275	MNKA 6631	Voucher	Padial and De La Riva 2009
*P. gutturalis*	French Guiane	JN690705	577PG	Voucher	Fouquet et al. 2012
*P. zeuctotylus*	Suriname	JN691256	1069BPN	Voucher	Fouquet et al. 2011
*P. achatinus*	Colombia	JN104676	UVC15867	Voucher	Garcia et al. 2012
*P. conspicillatus*	Ecuador	EF493529	QCAZ28448	Voucher	Heinicke et al. 2007
*P. skydmainos*	Peru	EF493393			Heinicke et al. 2007
*P. vilarsi*	Colombia	KP149438	AJC 3945	Voucher	Guarnizo et al. 2015
*P. samaipatae*	Bolivia, Santa Cruz	EU192292	MNCN 42987	Voucher	Padial and De La Riva 2009
*Oreobates cruralis*	Bolivia	JF809994		Voucher	Padial et al. 2012

Abbreviations: MNCN, Museo Nacional de Ciencias Naturales (Spain); url="http://grbio.org/institution/museo-de-historia-natural-noel-kempff-mercado-universidad-aut%C3%B3noma-gabriel-ren%C3%A9-moreno">MNH-A, Museum of Natural History Noel Kempff Mercado (Bolivia); MNH, Museum of Natural History, Universidad Nacional de San Antonio Abad del Cusco, Peru; INPA – H, Instituto Nacional de Pesquisas da Amazônia – Herpetologia; MPEG, Museu Paraense Emílio Goeldi; SISTAP, Sisbiota Tapajós; LZATM, Laboratório de Zoologia de Altamira.

The sequencing reaction was performed according to the manufacturer’s recommendations for sequencing mix ABI *BigDye Terminator*, using the primer 16Saf at an annealing temperature of 50 °C. The sequencing reactions were precipitated using the standardized protocol EDTA/Ethanol, resuspended with 10 μL deionized formamide (ABI) and sequenced in the automatic sequencer ABI 3130xl (*Applied Biosystems*).

Sequences were aligned using the ClustalW algorithm (Thompson et al. 1996) implemented in the software BioEdit 7.2 (Hall 1999). We used the software jModeltest 2.1.10 under the corrected Akaike information criterion to find the best evolutionary model. A maximum likelihood analysis was performed with the Treefinder software (Jobb 2008) using default settings and with 10000 bootstrap replicates. The Bayesian phylogenetic analysis using the evolutionary substitution model (GTR+G) was implemented in MrBayes v.3.2.6 software (Altekar et al. 2004), with the default heating values for two out of four chains, running 10^6^ generations, with tree sampling every 2000 generations. The “burn in” value was selected by visualizing the log likelihoods associated with the posterior distribution of trees in the software Tracer v 1.5 (Rambaut et al. 2014). We assessed convergence by examining the average standard deviation of split frequencies among runs (< 0.01). All trees generated before the flattening of the log likelihood curve were discarded. In all analyses, 10% of the samples were discarded as burn-in. The number of independent samples was considered sufficient when stationarity was reached and the effective sample sizes (ESS) were greater than 200. Uncorrected pairwise distances (*p*-distances) among *Pristimantis latro* sp. n. and other species of the *P. conspicillatus* group were calculated using MEGA 6.0 (Tamura et al. 2007). This analysis used sequences of several species belonging to the *Pristimantis conspicillatus* group (Padial et al. 2014) that are morphologically similar to *Pristimantis fenestratus* (Padial and De La Riva (2009). Hedges et al. (2008) indicates *Oreobates* as basal group of *Pristimantis*, therefore this genus was used as outgroup in our analyses. All sequences generated and/or analyzed in this study are available in GenBank (accession numbers are listed in Table 1).

## Results

### Phylogenetic analysis and genetic distances

**Figure 2.**
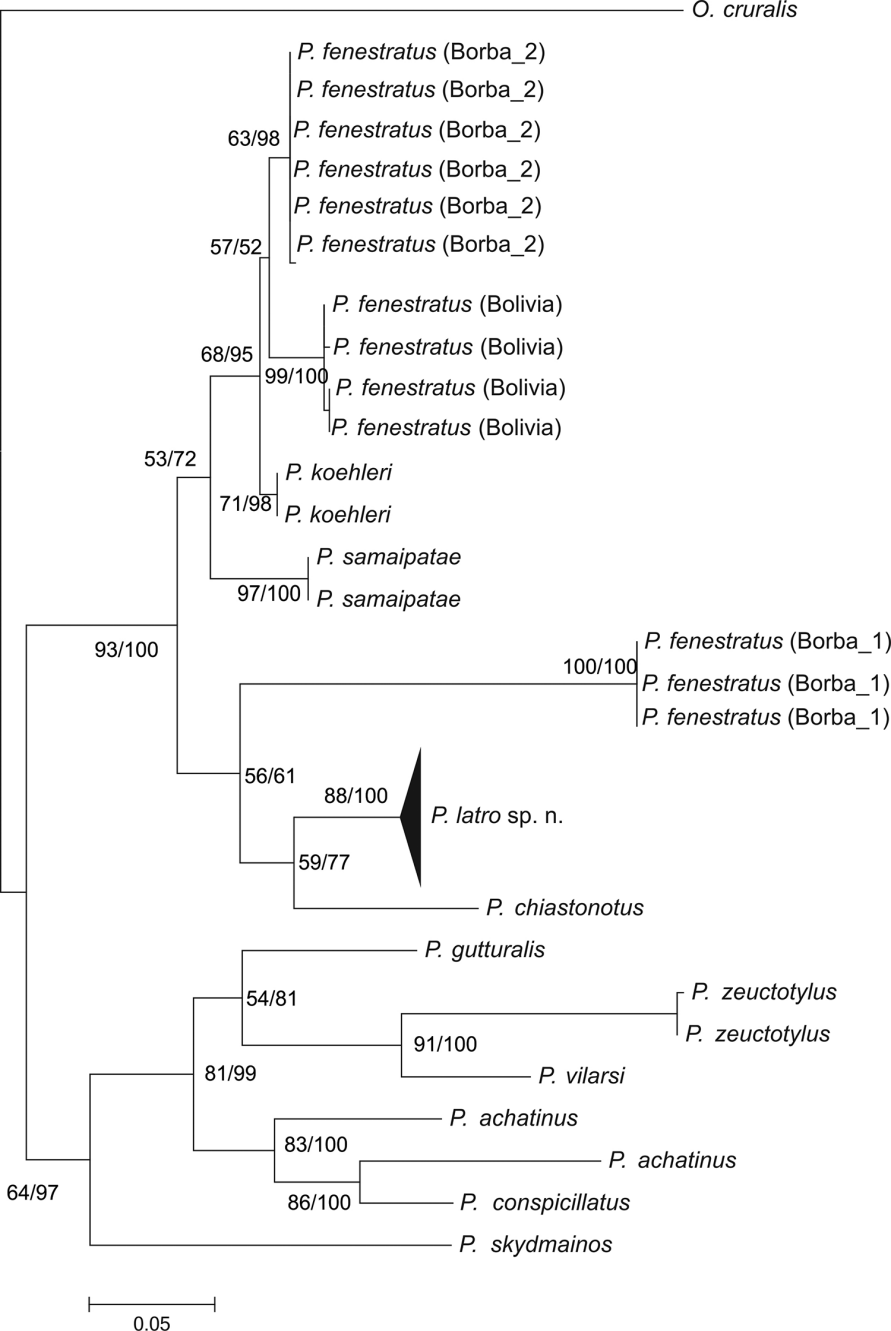
Maximum Likelihood (ML) tree using the evolutionary model GTR + G, inferring phylogenetic relationships of *Pristimantis* sp. n. and other species of the *P. conspicillatus* group based on mitochondrial 16S mtDNA (490 bp). ML support values are shown before the “/”. Bayesian posterior probability support values (%) for major respective nodes are shown after the “/”. The horizontal bar below the tree represents the genetic distance between branches. The branch of the new species was collapsed (black triangle) to improve tree visualization.

The phylogenetic analysis of the nominal species *Pristimantis fenestratus* revealed the existence of four lineages (Figure 2): two present in the municipality of Borba, Amazonas, Brazil; a third one for specimens from Bolivia available on GenBank, and a fourth lineage – the new species – that groups together individuals of the Xingu and Tapajos rivers in Pará, Brazil. Samples collected in the paralectotype locality of *P. fenestratus*, municipality of Borba, presented two lineages (Borba 1 and Borba 2) with a genetic distance of 13%. The individuals of Borba 2 (INPA-H 34571, 34577, 34562, 34573, 34578 and 34575) presented a genetic distance of 3% to *P. fenestratus* (Bolivia) and of 2% to *Pristimantis koehleri*. The individuals of Borba 1 (INPA-H 34565, 34579 and 34580) presented a genetic distance of 15% to *P. fenestratus* (Bolivia) and 13% to *P. koehleri*. The new species, *Pristimantis latro* sp. n. has a genetic distance of 8% to *P. chiastonotus*, 9% to Borba 2, 10% to *P. koehleri*, and 11% to both Borba 1 and *P. fenestratus* of Bolivia (Table 2).

**Table 2.** Genetic uncorrected pairwise distances (%) among species of the Pristimantis conspicillatus group and the outgroup considered in this study. The numbers at the top of the table correspond to the locations in the first column.

	1	2	3	4	5	6	7	8	9	10	11	12	13	14	15	16	17	18
*P. latro* (Anapu) - 1																		
*P. latro* (Senador) - 2	0.01																	
*P. latro* (Altamira) - 3	0.01	0.00																
*P. latro* (Medicilândia) - 4	0.01	0.00	0.00															
*P. latro* (Flona Tapajós) - 5	0.01	0.01	0.02	0.02														
*P. fenestratus* (Borba 1) - 6	0.11	0.11	0.11	0.11	0.10													
*P. fenestratus* (Borba 2) - 7	0.09	0.10	0.10	0.09	0.10	0.12												
*P. fenestratus* (Bolivia) - 8	0.09	0.09	0.09	0.09	0.10	0.13	0.03											
*P. chiastonotus* (Brazil) - 9	0.08	0.07	0.08	0.08	0.09	0.13	0.10	0.09										
*P. koehleri* (Bolivia) - 10	0.10	0.09	0.10	0.10	0.09	0.11	0.02	0.03	0.10									
*P. samaipatae* (Bolivia) - 11	0.09	0.09	0.10	0.10	0.09	0.11	0.05	0.06	0.08	0.04								
*P. gutturalis* (French Guiana) - 12	0.14	0.14	0.15	0.15	0.14	0.17	0.14	0.14	0.17	0.13	0.13							
*P. zeuctotylus* (Suriname) - 13	0.14	0.15	0.15	0.15	0.14	0.16	0.16	0.15	0.16	0.15	0.13	0.11						
*P. achatinus* (Colombia) - 14	0.15	0.14	0.14	0.14	0.15	0.17	0.15	0.15	0.16	0.14	0.15	0.12	0.14					
*P. conspicillatus* (Ecuador) - 15	0.13	0.13	0.14	0.14	0.14	0.14	0.13	0.13	0.14	0.13	0.12	0.10	0.11	0.09				
*P. skydmainos* (Peru) - 16	0.18	0.18	0.18	0.18	0.19	0.19	0.15	0.15	0.16	0.14	0.15	0.15	0.14	0.15	0.13			
*P. vilarsi* (Colombia) - 17	0.12	0.12	0.13	0.13	0.12	0.16	0.15	0.15	0.14	0.15	0.13	0.10	0.09	0.13	0.11	0.14		
*Oreobates cruralis* (Bolivia) - 18	0.17	0.18	0.19	0.19	0.18	0.22	0.18	0.17	0.20	0.19	0.19	0.17	0.19	0.20	0.19	0.20	0.19	

### Bioacoustic analysis (Figure 3)

The call is characterized as ascending: its first note has a dominant frequency of 2635 Hz and the last one of 3272 Hz. The number of recorded notes of all specimens was seven, with a length from 31.60 to 45.91 ms (average = 39.68 ± 5.12). Total duration of the call averaged 454.83 ms (± 68.99, 402.36–581.27), presenting multiple pulses per note (6–9, average = 7.5 ± 2.12). The fundamental frequency ranged from 1342 to 1448 Hz (average= 1381.41 ± 35.71) and the dominant frequency ranged from 2635 to 3272 Hz (average= 3069.21 ± 253.61). A comparison between the advertisement call parameters of *Pristimantis latro* sp. n. and other species of *Pristimantis conspicillatus* group is shown in Table 3.

**Figure 3.**
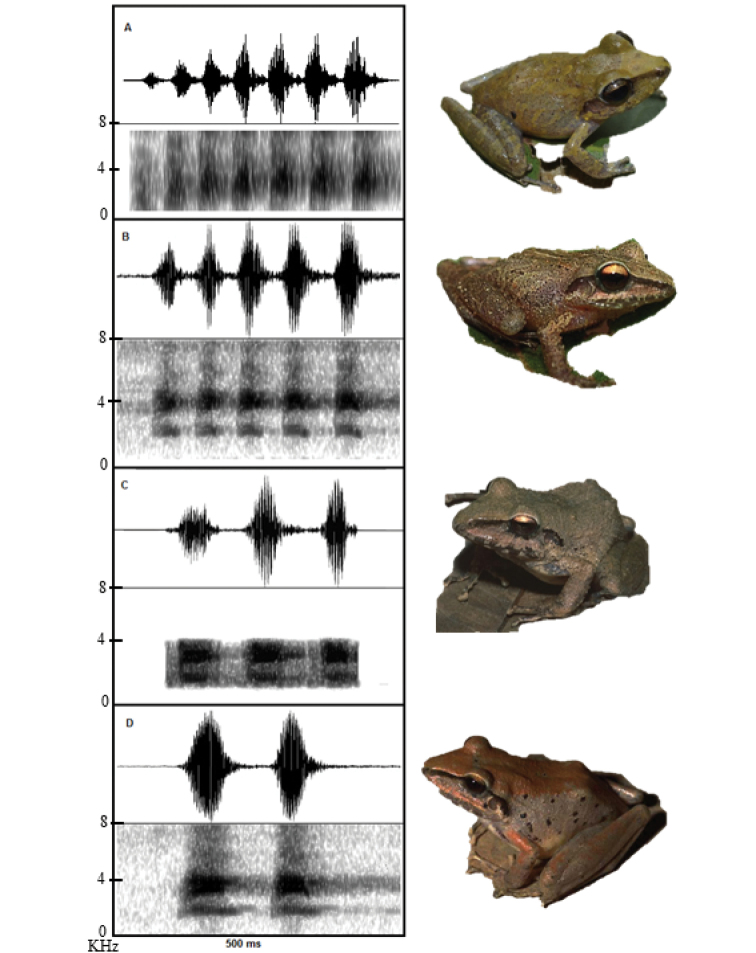
Comparison between advertisement calls among some species of the *Pristimantis conspicillatus* group: **A** *P. latro* sp. n. **B** *P. koehleri* **C** *P. fenestratus* and **D** *P. samaipatae*.

**Table 3.** Diagnostic characters of advertisement calls from species of the *Pristimantis conspicillatus* group. Values are given as range (average ± standard deviation).

Species	Notes/Call	Call length (ms)	Note length (ms)	Pulses	Fundamental frequency (Hz)	Dominant frequency (Hz)	Notes	Calls	N specimens	N populations	Source
*P. fenestratus*	2–4 (2.6 ± 0.6)	157–458 (265.2 ± 81.6)	50–91 (63 ± 11.4)	9–17 (12.9 ± 42.2)	1542–2048 (1746 ± 158)	1710–3591 (3086.3 ± 580.7)	55	22	6	4	Padial and De La Riva (2009)
*P. koehleri*	3–8 (5.7 ± 1.0)	173–644 (421 ± 159.8)	20–54 (35.5 ± 6.6)	5–9 (7.5 ± 1)	1732–1971 (1853.5 ± 72.1)	3245–3971 (3662.4 ± 128.9)	119	21	6	2	Padial and De La Riva (2009)
*P. samaipatae*	1–3 (2 ± 0.2)	82.2–1062 (291.7 ± 168.1)	59–141 (89 ± 16.4)	11–23 (16.4 ± 2.6)	1535–1834 (1704.9 ± 64.3)	2922–3853 (3326.7 ± 175.9)	160	98	12	4	Padial and De La Riva (2009)
*P. latro*	**7**	**402.36–581.27** (454.83 ± 68.99)	**31–45.918 (39.686 ± 5.12)**	**6–9 (7.5 ± 2.12)**	**1342–1448,6 (1381.41 ± 35.71**	**2635.89–3272 (3069.21 ± 253.61)**	**49**	**7**	**6**	**2**	**This study**

### Morphological analysis

Based on qualitative morphological characters, the new species can be distnguished from other species of the *conspicillatus* group from the state of Pará by having divided palmar tubercle and venter cream with black spots, while *P. zeuctotylus* has undivided palmar tubercle and black venter. When compared with *P. chiastonotus*, the new species differs by the presence of a basal webbing among toes and the presence of a tarsal fold, absent in *P. chiastonotus*. When compared to *P. fenestratus*, lineage Bolivia, the new species lacks discoidal folds and presents a supernumerary tubercle in the hand. Additional details can be found in the section "Comparasion with other species".

With regard to quantitative morphological traits, males of the new species have a smaller SVL (N = 46, mean = 27.4 ± 7.2) compared to other lineages of *Pristimantis fenestratus* (Bolivia, N = 44, mean = 30.5 ± 2.1, from Padial and De La Riva 2009), lineage Borba 1 (N = 5, mean = 31.2 ± 1.9), lineage Borba 2 (N = 3, mean = 31.0 ± 0.2), and *P. chiastonotus* (N = 20, mean = 33.0). Only *P. zeuctotylus* has a smaller SVL than the new species (N = 20, mean = 25.2). Due to the low number of females form the localities Borba 1 and Borba 2, we restrict our comparisons with *P. fenestratus* from Bolivia (Padial and De La Riva (2009), *P. chiastonotus* and *P. zeuctotylus*. Females of the new species have a smaler SVL (N = 49, mean = 31.2 ± 7.9) than *P. fenestratus* from Bolivia (N = 44, mean = 43.7 ± 4.6), *P. chiastonotus* (N = 14, mean = 44.0) and *P. zeuctotylus* (N = 32, mean = 37.0). All measurements from the new species can be found on Appendix 2.

#### Pristimantis latro sp. n.

Taxon classificationAnimaliaAnuraCraugastoridae

http://zoobank.org/19BF72F8-BDA4-4C8C-965D-0D92B654B1DA

Figure 4

##### Holotype.

LZATM – 467, adult female, collected on July 23, 2012 in the municipality of Anapu, Pará State, Brazil (3°9'28.15"S; 51°27'51.67"W) by Elciomar Araújo de Oliveira, Emil José Hernández Ruz and Joyce Celerino de Carvalho. Material stored in the collection of the Laboratório de Zoologia de Altamira (LZATM) of the Universidade Federal do Pará, Campus de Altamira, Brazil.

*Paratopotypes.* Two adult males: LZATM 739, LZATM 747 and nine adult females: LZATM 743, LZATM 749, LZATM 750, LZATM 740, LZATM 742, LZATM 754, LZATM 742, LZATM 748, LZATM 751, collected during field work by Claudia Liz Teles and Joyce Celerino de Carvalho. Material stored in the collection of the Laboratório de Zoologia de Altamira (LZATM) of the Universidade Federal do Pará, Campus de Altamira, Brazil.

*Paratypes*. Six males: LZATM 197, LZATM 0063, LZATM 1339, LZATM 818, LZATM 815, LZATM 816 and LZATM 1340. Eleven females: LZATM 386, LZATM 243, LZATM 360, LZATM 744, LZATM 281, LZATM 742, LZATM 748, LZATM 751, LZATM 230, LZATM 358 and LZATM 277 collected during field work by Claudia Liz Teles and Joyce Celerino de Carvalho. Material stored in the collection of the Laboratório de Zoologia de Altamira (LZATM) of the Universidade Federal do Pará, Campus de Altamira, Brazil. The collection locations of each specimen are listed in Appendix 1.

**Figure 4.**
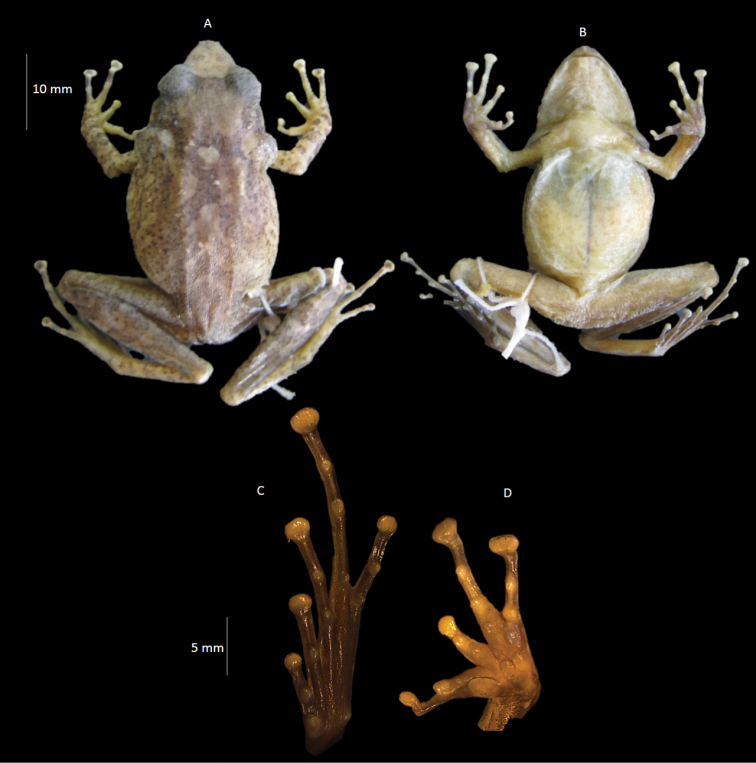
Holotype of *Pristimantis* sp. n. **A** ventral view **B** dorsal view **C** side view of the head **D** hand **E** and right foot (INPA-H 34576).

##### Allocation to genus and species group of the new species.

No morphological synapomorphy has yet been identified to support the genus *Pristimantis* (Hedges et al. 2008). The new taxon is therefore assigned to the genus *Pristimantis* based on (1) molecular phylogenetic relationships (Figure 2); and (2) its morphological characteristics, which fall into the range of other known *Pristimantis* species. The new taxon is assigned to the genus *Pristimantis* based on its geographic distribution and overall similarity to the majority of species of *Pristimantis* described. We assign the new species to the *P. conspicillatus* species group following Maciel et al. (2012) for having Finger I longer than Finger II, granular but nor aerolate belly, a tarsal fold, distinct tympanic membrane, and by its advertisement call composed of single pulsatile notes modulated in amplitude, as well as molecular phylogenetic relationships.

##### Diagnosis.

*Pristimantis latro* sp. n. is distinguished from other species of the group by the following combination of characters (summarized in Table 4): (1) dorsal skin weakly shagreened or smooth, dorsal tubercles present, dorsolateral folds absent, smooth skin on belly; (2) whitish or yellowish ventral coloration with black spots; (3) one subarticular tubercle on fingers I and II and two on Fingers III and IV; (4) supernumerary tubercles present at the base of fingers I, II, and III, and almost the same size of subarticular tubercles; (5) lateral fringes along fingers; (6) supernumerary tubercle present at the base of Toe IV; (7) basal webbing between toes and weak lateral fringes on toes; (8) twenty-one molecular autapomorphies for the gene fragment 16S mtDNA (Table 5); (9) call consisting of seven notes.

**Table 4.** Comparison of diagnostic characters of some species of the *Pristimantis conspicillatus* group, including the new species: (1) belly texture (smooth or granular), (2) dorsal tubercles (present or absent); (3) fringe on finger (present or absent); (4) dorsolateral fold (present or absent); (5) fringe on toe (prominent, weak, absent); (6) basal membrane on toe (present or absent); (7) tarsal fold (prominent, weak or absent); (8) throat color pattern (stained, immaculate, variable or light); (9) supernumerary plant tubercle (present or absent); (10) External palmar tubercle (whole, split or semi-split).

Species	1	2	3	4	5	6	7	8	9	10
P. fenestratus*	smooth	present	absent	–	weak	present	–	stained	absent	–-
P. fenestratus**	smooth	absent	present	absent	weak	present	present	variable	–	split
*P. koehleri*	granular laterally	absent	absent	absent	weak	absent	present	light	present	split
*P. dundeei*	granular	present	absent	absent	prominent	present	present	stained	–	split
*P. samaipatae*	smooth	absent	absent	absent	prominent	absent	present	stained	–	split
*P. ventrigranulosus*	granular	absent	weak or absent	absent	weak	present	prominent	weakly spotted	absent	single
*P. zeuctotylus*	smooth	absent	absent	present	absent	absent	absent	stained	present	inteiro
*P. chiastonotus*	smooth	absent	absent	present	absent	absent	absent	ligth	present	split
*Pristimantis latro* sp. n.	**smooth**	**present**	**present**	**present**	**weak**	**present**	**weak**	**stained**	**present**	**split**

**Table 5.** Diagnostic characters observed in the 16S mtDNA gene fragment from *Pristimantis* sp. n. and other species of the genus *Pristimantis*. The first column indicates the character position within the fragment. (-) indicates deletions.

Position (pb)	*P. latro* sp. n.	*P. fenestratus* (Borba 1)	*P. fenestratus* (Borba 2)	*P. fenestratus* (Bolivia)	*P. koehleri* (Bolivia)	*P. chiastonotus* (Brazil)
86	G	A	A	A	A	A
138	A	G	G	G	G	A
144	T	C	C	C	C	C
149	A	T	T	T	T	A
184	T	C	C	C	C	C
194	C	-	-	-	-	A
197	T	A	A	A	A	C
202	T	A	A	A	A	-
208	T	C	C	C	C	C
229	C	T	A	A	A	T
230	C	T	T	T	T	T
237	T	-	-	-	-	C
239	T	C	C	C	C	A
247	C	A	T	T	T	-
269	C	T	T	T	T	T
273	A	C	T	T	T	A
289	G	A	A	A	A	A
293	T	-	A	A	A	-
330	C	T	T	T	T	T
401	G	A	A	A	A	A
455	C	T	T	T	T	A

##### Comparison with other species.

Due to difficulties in visiting museums to compare some of the species in the *Pristimantis conspicillatus* group with the species described in this work, data from the literature was used for this procedure. The consulted reference can be found, between brackets, at the end of each comparison. The character state of the compared species is between parentheses. *Pristimantis latro* sp. n. is distinguished from *P. fenestratus* by the absence of discoidal fold (present), the presence of supernumerary tubercles on hand (absent), length of notes in the male advertisement call ranging from 31 to 45.91 ms (50 to 91 ms) [Duellman and Lehr 2009; Padial and De La Riva 2009; Maciel et al. 2012]; from *P. koehleri* by smooth belly skin (finely granular), absence of discoidal fold (present), rostrum subacuminate in dorsal and protruding in lateral view (acuminate in dorsal view and subacuminate in lateral view), vocalization composed by seven notes (four, five, six, seven and eight notes) [Padial and De La Riva 2009]; from *P. samaipatae* by having whitish cream belly with black spots disposed randomly (immaculate), length of notes ranging from 31 to 45.91 ms (50 to 141 ms in *P. samaipatae*) [Köhler 2000; Padial and De La Riva 2009]; from *P. dundeei* by having smooth belly (areolate), presence of fringe in the fingers (absent), dorsolateral folds (absent), length of notes ranging from 31 to 45.91 ms (50 ms in *P. dundeei*) [Köhler 2000; Padial and De La Riva 2009]; from *P. ventrigranulosus* by having smooth belly skin (weakly areolate), dorsal tubercles (absent), presence of fringe in the fingers (weak or absent), dorsolateral fold present (absent), weak tarsal fold (prominent) [Maciel et al 2012]; from *P. zeuctotylus* by a divided palmar tubercle (entire), whitish cream-colored belly with black spots disposed randomly and dark brown dorsum (black belly and bronze dorsum) [Lynch and Hoogmoed 1977]; from *P. chiastonotus* for presenting basal webbing and fringe on the toes (absent), tarsal fold present (absent); snout subacuminate in dorsal view (acuminate), dorsal tubercles present (absent), vocalization composed by seven notes (one note) [Lynch and Hoogmoed 1977].

The comparisons were restricted to these species because they present the highest morphological and acoustic similarity with the new species. Another important factor is the geographical range of the new species, which becomes the only one in its group occurring in the eastern state of Pará, Brazil. The geographically-closest species are *P. zeuctotylus* and *P. chiastonotus*, north of Pará, whereas the most genetically-close are *P. chiastonotus* from the municipality of Monte Alegre in the state of Pará and the lineage of *P. fenestratus* from Borba 1 in the state of Amazonas.

##### Description of the holotype.

Adult female 40 mm SVL. Dorsal skin shagreened, absence of dorsal tubercles; smooth ventral skin, granular posterior surface of thighs; head longer (39% of the SVL) than wide; long snout, subacuminate in dorsal view and protruding in lateral view; concave canthus rostralis, flat loreal region; ovoid tongue covering the whole floor of the mouth; dentigerous process of vomer oblique and posterior to choanae; eye 78.9% of Distance from Eye to Nostril; elliptical pupil; absent supraocular tubercles; absent cranial crests; prominent supra tympanic fold, not contacting the eye; tympanic membrane 40% of ED, rounded, tympanic annulus prominent; relatively small hands, 26.25% of the SVL; relative length of fingers: II < IV < I < III; discs of Fingers III and IV are wider than fingers I and II; prominent, semi divided, heart-shaped external metacarpal tubercle; large internal palmar tubercle; one subarticular tubercle prominent on Fingers I and II, two prominent subarticular tubercles on fingers III and IV; supernumerary tubercles present at the base of fingers I, II and III; long legs, tibia 57% of the SVL; relative length of toes: I <II <V <III <IV; well developed and oval inner metatarsal tubercle; external metatarsal tubercle much smaller than the internal one; one subarticular tubercle on toes I and II; two subarticular tubercles on toes III and V; and three subarticular tubercles on toe IV; basal webbing and lateral fringes present on toes (weak); tarsal fold present.

*Measurements of holotype* (in mm). SVL: 40.0; HL: 15.6; HW: 14.5; SL: 7.9; DEN: 5.7; ID: 3.1; EL: 4.5; IoD: 3.9; EW: 3.6; TL: 1.8; AL: 8.9; HaL: 10.5; ThL: 20.5; TiL: 22.8; TaL: 11.9; FL: 18.9; LL: 30.3.

*Color in life.* Light brown dorsum with some black tubercles. Posterior and anterior limbs heavily barred dark brown. Weak labial bars. Black band extending from eye to tip of snout. Belly clear with some randomly scattered dark spots. Iris presents a yellowish coloration in the upper and lower part, whereas in the anterior and posterior region the color red is predominant.

*Coloration in preservative.* In alcohol, the coloration is predominantly brown in the dorsal region, whether male or female. The belly can be immaculate white or present dark spots arranged randomly. The dorsal band, present in some individuals, is white.

**Figure 5.**
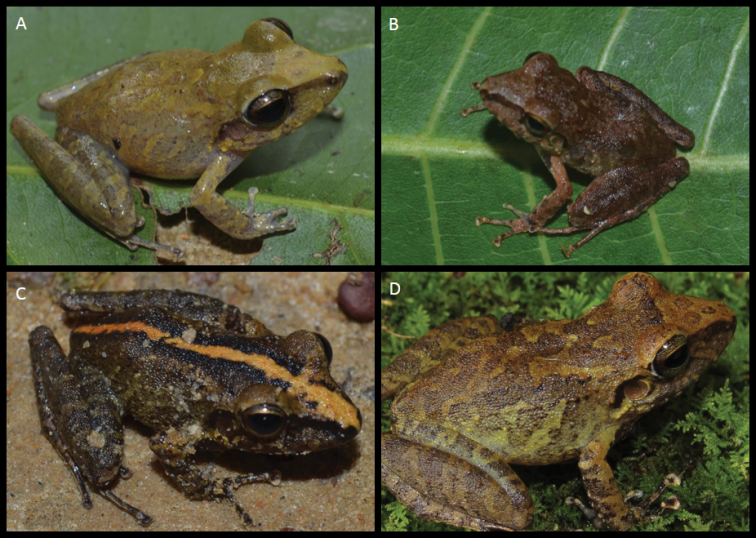
Color variation in life of some individuals of *Pristimantis latro* sp. n. **A** holotype **B, C** paratypes of Anapu and **D** Altamira.

**Figure 6.**
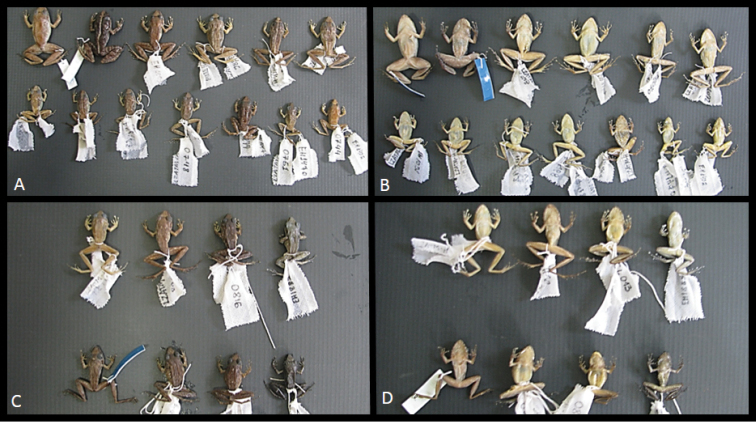
Dorsal and ventral morphological variation of the type series of *Pristimantis latro* sp. n. **A** Females in dorsal view **B** Females in ventral view **C** Males in dorsal view and **D** Male in ventral view.

*Variation* (Figures 5 and 6). The males LZATM 197, LZATM 063 and LZATM 1339 have dorsal color light brown, while the males LZATM 818, LZATM 815, LZATM 816 and LZATM 1340 have dark brown dorsal and dorsolateral regions with more apparent brown bars. The ventral face of males may be immaculate white (LZATM 197, LZATM 816) or have black spots scattered around the belly and throat (LZATM 1339, LZA 063, LZATM 818, LZATM 815 and LZATM 1340). LZATM 1340 presents a heavily pigmented black throat, legs and arms with clear bars. Females have predominantly light brown dorsum, with weakly barred legs and arms of darker brown (LZATM 386, LZATM 467, LZATM 243, LZATM 360, LZATM 744, LZATM 281, LZATM 742, LZATM 748 and LZATM 751), while LZATM 230 and LZATM 358 have a darker coloration and a dorsal band from the face to the cloaca of yellow color (in life) and white (in alcohol). The latter individual has strongly barred legs and arms. Its belly is usually either immaculate white or with a few dark spots, but LZATM 277 has a belly and throat heavily black pigmented. The dorsal skin is smooth in most of the examined individuals, although some specimens present a weakly shagreened texture: LZATM 358, LZATM 816, LZATM 63, LZATM 1339, LZATM 1340 and LZATM 467.

##### Etymology.

The specific epithet “latro” (from the Latin *latro* = mercenary, robber) refers to the common name generally attributed to the species of *Pristimantis* – “Robber Frogs” – that exhibit a dark band on the snout, creating the illusion of a robber’s mask.

##### Distribution, ecology, and habitat.

**Figure 7.**
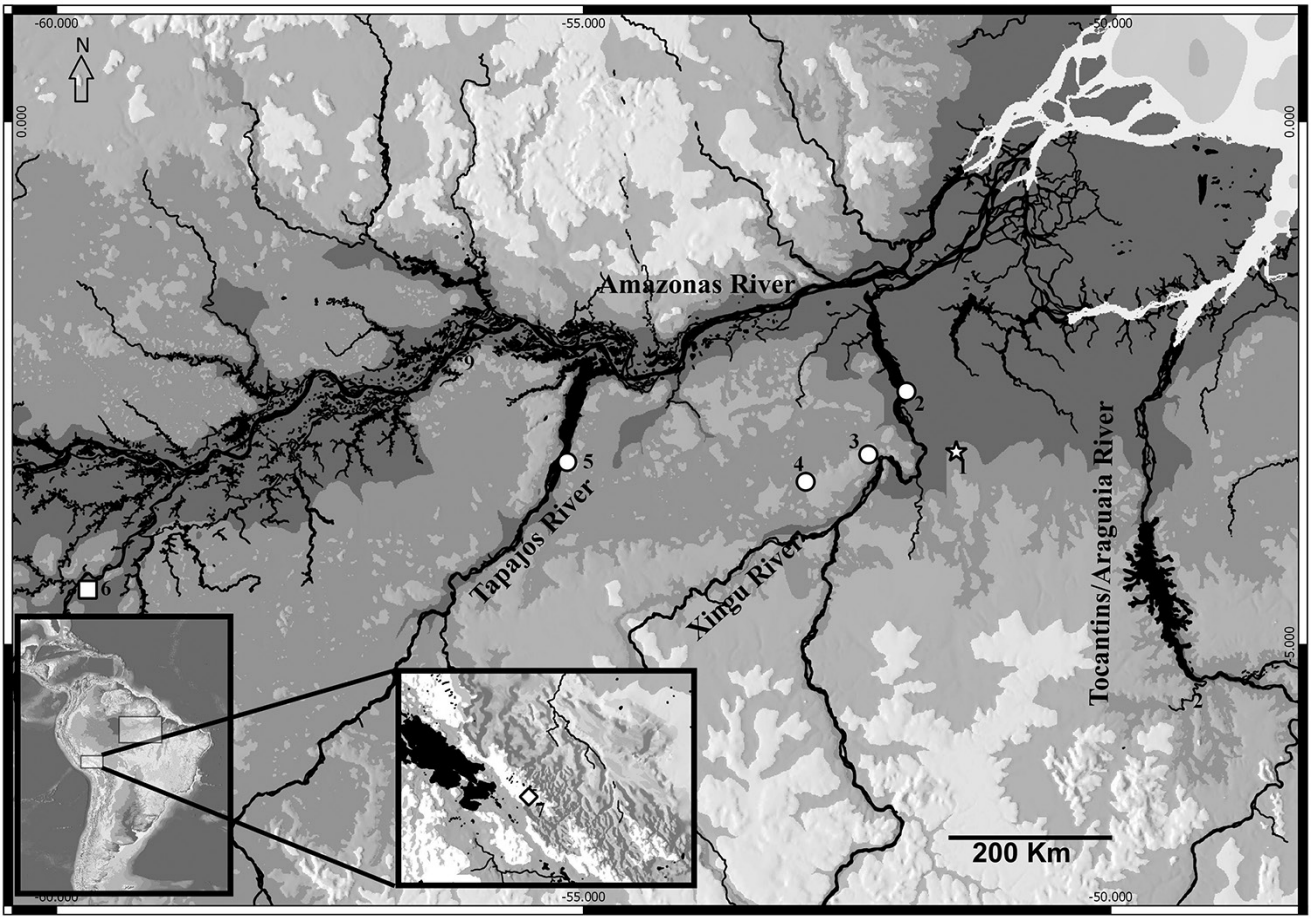
Type locality of *Pristimantis latro* sp. n., municipality of Anapu, Pará, Brazil (star). The circles represent the other localities where the new species was found. The square and the diamond represent the localities of *Pristimantis fenestratus* used for the morphological and genetic comparisons. **1** Anapu (3°4'57.26"S; 51°22'25.67"W) **2** Senador José Porfírio (2°34'51.63"S; 51°56'13.47"W) **3** Altamira (3°13'24.85"S; 52°14'22.74"W) **4** Medicilândia (3°26'37.93"S; 52°53'35.26"W) **5** Flona do Tapajós (3°38'49.06"S; 55°11'46.00"W) **6** Borba (4°28'29.88"S; 59°42'12.06"W) and **7** La Paz, Bolivia (16°24'12.89"S; 68°6'10.20"W).

*Pristimantis latro* sp. n. has been recorded in the municipalities of Anapu, Senador Jose Porfirio, Altamira, Medicilândia, Brasil Novo, Uruará and Flona Tapajós regions located in the interfluves Xingu / Tapajós and Xingu / Tocantins - Araguaia in Pará State, Brazil (Figure 7). It can be found in conserved areas of forests (Anapu, Flona do Tapajós) or with some environmental disturbance, e.g., forest fragments surrounded by pastures (Brasil Novo, Altamira and Vitória do Xingu). During the rainy/reproductive period, the males move up the vegetation to vocalize at a height of 1.5 m and in the dry period they can be found in the leaf litter.

## Discussion

*Pristimantis* is a megadiverse genus with many species described mainly for the Andean region of Peru and Bolivia, Colombia, Ecuador and Venezuela, likely because a larger number of surveys have been carried out in these areas (Duellman and Hedges 2007, Elmer and Cannatella 2008, Padial and De la Riva 2009, Duellman and Lehr 2009, Barrios-Amorós et al. 2010, Arteaga-Navarro and Guayasamin 2011, Mueses-Cisneros et al. 2013, Navarrete et al. 2016, Shepack et al. 2016). In comparison, its diversity in the eastern Amazonian region appears to be lower, possibly due to a lack of taxonomic studies. As far as we know, *Pristimantis latro* sp. n. represents the first species described to the south of the Amazon River in Pará State, where it was erroneously identified as *P. fenestratus* due to their morphological similarities (Oliveira et al. 2013, Vaz-Silva et al. 2015).

*Pristimantis fenestratus* has been considered a widely distributed and recorded species in the Amazon, but we raise a problem already mentioned by other authors regarding its cryptic diversity (Padial and De la Riva 2009, Duellman and Lehr 2009, Smith et al. 2009). Our analyses show that *P. fenestratus* from the municipality of Borba and *P. fenestratus* from La Paz, Bolivia, represent three lineages separated by genetic distances larger than 3%, which studies suggest may indicate distinct species (Vences et al. 2005, Fouquet et al. 2007). Thus, a taxonomic revision of *P. fenestratus* is required since populations from the two locations mentioned in the original description show considerable genetic differences.

*Pristimantis latro* sp. n. is described for the Eastern Amazonia after a morphological, molecular and bioacoustics comparison with *P. fenestratus* and other species of the *P. conspicillatus* group. Recent studies have revealed that widely distributed frog species often include many cryptic taxa (Elmer et al. 2007, Padial and De la Riva 2009, Gehara et al. 2014). It is common to describe new species of *Pristimantis* based only on morphology (Barrios-Amorós et al. 2010, Mueses-Cisneros et al. 2013, Navarrete et al. 2016) or on a combination of morphological and genetic evidences (Arteaga-Navarro and Guayasamin 2011, Barrios-Amorós et al. 2012). Here, the combination of different lines of evidence revealed a new species of *Pristimantis* with morphological, genetic and bioacoustic diagnostic characters that allow not only differentiating it from other species of its group, but also illuminate the taxonomy of this speciose genus.

## Supplementary Material

XML Treatment for Pristimantis latro

## Appendix 1

**Specimens examined.** INPA-H (Instituto Nacional de Pesquisas da Amazônia – Herpetology); MPEG (Museu Paraense Emilio Goeldi); LZATM (Laboratório de Zoologia de Altamira); CTGANSISTA_D (Coleção de Tecidos de Genética Animal, Sisbiota Tapajós, margem direita).

*Pristimantis fenestratus*: INPA-H 34562, INPA-H 34565, INPA-H 34571, INPA-H 34573, INPA-H 34575, INPA-H 34577, INPA-H 34578, INPA-H 34579, INPA-H 34580, MPEG 7088, municipality of Borba, Amazonas, Brazil (type locality).

*Pristimantis* sp. n.: LZATM467, LZATM743, LZATM 739, LZATM749, LZATM747, LZATM750, LZATM740, LZATM742, LZATM754, LZATM742, LZATM748, LZATM751, MPEG 26050, MPEG 26059, MPEG 26052, MPEG 26063, MPEG 26065, municipality of Anapu, Pará, Brazil (type locality). LZATM 0063, LZATM139, LZATM155, LZATM213, LZATM265, LZATM270, LZATM277, LZATM280, LZATM281, LZATM386, LZATM 1344, LZATM 1112, MPEG 26055, MPEG 26053, MPEG 26054, MPEG 31415, MPEG 31416, MPEG 1113, municipality of Altamira, Pará, Brazil. LZATM137, LZATM138, LZATM197, LZATM 802, LZATM 876, LZATM 1340, municipality of Brazil Novo, Pará, Brazil. LZATM140, LZATM141, LZATM188, LZATM222, LZATM229, LZATM230, LZATM236, LZATM243, LZATM255, LZATM 818, LZATM 814, LZATM 816, LZATM 815, municipality of Medicilândia, Pará, Brazil. LZATM355, LZATM356, LZATM357, LZATM358, LZATM359, LZATM360, municipality of Uruará, Pará, Brazil. LZATM 753, LZATM 1125, LZATM 1140, municipality of Senador José Porfírio, Pará, Brazil. CTGANSISTA_D_1168, CTGANSISTA_D_1246, CTGANSISTA_D_1235, CTGANSISTA_D_1145, CTGANSISTA_D_1253, CTGANSISTA_D_1239, CTGANSISTA_D_1275, CTGANSISTA_D_1259, CTGANSISTA_D_1260, CTGANSISTA_D_1257, CTGANSISTA_D_1256, CTGANSISTA_D_1244, CTGANSISTA_D_1240 Flona Tapajós, Pará, Brazil.

*Pristimantis zeuctotylus*: LZATM1951, LZATM1054 and LZATM1057, municipality of Monte Alegre, Pará, Brazil.

*Pristimantis chiastonotus*: LZATM1050, LZATM1052, LZATM1055, and LZATM1056, municipality of Monte Alegre, Pará, Brazil.

## Appendix 2

**Table:** Morphological measurements of all Brazilian specimens of *Pristimantis latro* sp. n. examined in this study.

Locality	Exemplar	Sex	SVL	ThL	FL	HL	HW	IoD	WS	ID	DEN	EL	TL	LT	AL	SL	LL	TaL	HaL
Anapu – Brazil	LZATM 467	F	40	20.5	18.9	15.6	14.5	3.9	3.6	3.1	5.7	4.5	1.8	22.8	10.5	7.9	30.3	11.9	8.9
Anapu –	LZATM 743	F	19.1	10	8.3	7.5	6.5	2.1	2	1.9	2.9	2.5	1	11.6	5.2	3.9	14.3	6.2	4.3
Anapu –	LZATM 739	M	16.9	8.9	7.3	6.9	5.7	2	1.5	1.6	2.4	2.5	0.8	9.7	4.4	3.4	11.8	4.5	3.6
Anapu –	LZATM 749	F	40.2	20.4	19	16.2	15	3.3	3	3.7	5.8	4.7	2.1	22.8	10.1	8	29.4	10.6	8.8
Anapu –	LZATM 747	M	22.8	12.8	11	9.6	8.3	2.4	2.2	2	3.3	3.6	1.2	13.9	5.9	4.7	17.1	7	5.6
Anapu –	LZATM 750	F	21.7	11.5	10	8.7	7.6	2	2.1	1.9	3	3.1	1.1	12.7	5.5	4.7	16.4	6.8	4.7
Anapu –	LZATM 740	F	26.4	14.3	12.5	10.3	9.5	2.8	2.7	2.3	3.7	3.3	1.5	15.2	6.8	5.3	19.9	8.4	5.8
Anapu –	LZATM 744	F	26.4	14.5	11.7	10.7	9.4	2.2	2.4	2.1	3.8	3.2	1.2	15.7	6.9	5.6	20.4	8.4	6.2
Anapu –	LZATM 754	F	18.8	9.7	8.8	7.6	6.1	1.6	2.2	1.7	2.8	2.8	1	10.7	4.4	3.8	13.8	6.2	4.4
Anapu –	LZATM 742	F	26.8	14.5	13.9	10.6	9.8	2.5	2.9	2.4	3.9	3.2	1.4	16.6	7.3	5.3	21.9	8.9	5.9
Anapu –	LZATM 748	F	27.8	13.5	14.6	10.9	9.9	2.6	2.3	2.6	3.8	3.3	1.4	17.2	7.2	5.6	22.9	8.2	6.4
Anapu –	LZATM751	F	24.4	12.3	11.8	9.2	7.9	2.2	2.4	2.2	3.1	3.1	1.1	13.8	6.1	4.5	18	7.2	5.5
Anapu –	MPEG 26050	F	41.6	21.7	19.4	16.1	15.8	3.7	4.4	3.7	5.7	5.8	2.5	24.5	11.3	8.2	29.8	11.4	8.9
Anapu –	MPEG 26059	F	38	17.5	17.5	14.5	13.7	3.7	4.4	3.1	5.3	5.0	2.3	20.3	9.2	7.8	27	10	7.5
Anapu –	MPEG 26052	M	27	12.1	12.8	9.8	9.2	2.6	2.6	2.3	3.6	3.6	1.4	14.5	7	5.1	19.3	7.3	6
Anapu –	MPEG 26063	F	19.5	10.5	9.2	7.8	7	2.1	2.5	2.1	2.8	3.1	1	11.6	4.9	4	15.4	6	4.8
Altamira – Brazil	LZATM63	M	28.3	14.8	14.8	11.6	10.1	2.7	2.9	2.6	4.1	3.9	1.6	16.8	7.6	5.8	22.9	8.4	6.9
Altamira –	LZATM139	F	38.3	20.4	14.8	15	13.6	3.4	3.3	3	5.5	4.4	2	23.1	10	7.6	28.1	12	8.4
Altamira –	LZATM155	F	28.2	14.1	13.4	10.4	8.4	1.9	3.2	2.3	3.9	3.2	1.4	16.1	7.2	5.4	21.3	8.9	6.5
Altamira –	LZATM213	F	36.2	18.5	17	13.6	13.6	2.7	3.5	2.9	4.8	4	1.9	21.1	8.8	6.3	27.4	10.7	8.3
Altamira –	LZATM 265	F	26.2	13.9	13.9	10.5	9.4	2.7	2.2	2.2	3.9	3.8	1.4	16	7.2	5	21.4	7.8	6
Altamira –	LZATM270	F	32.4	18	18.4	13	11.8	3	3.1	2.8	4.8	4	2	21.2	9.7	6.2	28.5	10	8.5
Altamira –	LZATM277	F	29.7	15.4	13.9	11.7	10.6	2.7	2.9	2.5	4.3	3.5	1.7	16.3	6.8	6.1	21.8	8.5	6.4
Altamira –	LZATM279	F	28.9	15.5	13.2	14.9	10.2	2.3	3.6	2.6	3.9	3.4	1.6	15.7	7.1	5.2	29.6	8	6.4
Altamira – Brazil	LZATM280	F	24.8	13.2	12.5	10.4	10	2.3	2.8	2.3	3.5	3.4	1.2	15.1	6.8	5	19.7	8	5.8
Altamira –	LZATM281	F	26.2	14.3	12.8	10.3	9.4	2.8	2.6	2.3	3.6	3.3	1.3	15.7	6.6	5	20.4	8	6
Altamira –	LZATM386	F	36.2	19.1	19.2	14.4	13.4	3.7	3.5	3.1	5.5	4.4	1.8	21.7	10.6	7.4	28.9	10.5	8.5
Altamira –	LZATM622	M	15.6	8	6.3	6	5.4	1.7	1.6	1.6	2	2.1	0.8	8.8	4	2.9	10.6	4.5	3.5
Altamira –	MPEG 31415	M	23.2	10.8	10.9	8.8	8.2	2.4	2.6	2.2	3	3	1.2	12.5	5.7	4.5	16.3	6.2	4.8
Altamira –	MPEG 31416	M	26.2	12.2	12.8	10.1	9.8	2.7	3.1	2.3	3.4	4	1.6	14.8	6.7	5.1	19.5	7.5	5.5
Brazil Novo – Brazil	LZATM802	F	36.2	18.8	19.3	13.1	11.9	3.6	3.2	2.9	4.8	4.4	1.7	21.6	10.2	7.5	29.8	11	8.4
Brazil Novo –	LZATM137	F	35.5	18.5	16.4	14	13.8	2.9	2.7	2.7	5.1	4	1.4	19.9	8.9	7	25.7	9.5	7.7
Brazil Novo –	LZATM138	F	38.2	20.3	18	15.3	14.4	3.3	3.3	3.3	5.4	4.6	2.2	21.8	9.6	7.7	28.7	10.9	8.8
Brazil Novo –	LZATM197	M	25	12.9	12.4	10	9.4	2.7	2.1	2.3	3.7	3.4	1.4	15.7	6.8	5.2	19.9	8.1	6.3
Medicilândia – Brazil	LZATM140	F	30.1	14.9	16.4	11.9	10.7	2.5	2.9	2.7	4.3	3.5	1.4	18.8	8.5	6.2	24.8	8.9	7
Medicilândia –	LZATM141	F	35.9	18.1	18.8	13.3	12.5	3.4	3.1	2.9	5	4.4	1.5	22.7	10.3	7.3	29.7	11.2	8.3
Medicilândia –	LZATM188	F	37.3	18.8	19.2	14	13	3	3.2	2.7	5.1	4.5	1.9	22.4	9.1	6.6	30	11.4	8.7
Medicilândia –	LZATM222	F	35.8	13.9	14	9.8	9.4	2.3	2.2	2.1	3.4	3.1	1.4	16	7	5	21.2	7.9	5.9
Medicilândia –	LZATM229	F	22.2	11.4	11.2	8.8	8.9	2	2	2	3.1	2.8	1.2	14	5.9	4.4	18.4	7.5	5
Medicilândia –	LZATM230	M	25.6	13.8	13	10.1	9.2	2.4	2.2	2.2	3.4	3.4	1.4	15.3	6.7	4.7	21.1	7.8	5.9
Medicilândia –	LZATM236	M	25	13.5	13	10.2	9.1	2.8	2.3	2.1	3.7	3.3	1.5	15.8	7	4.9	20.5	7.4	6.2
Medicilândia –	LZATM243	M	36	18.9	18.5	14.1	13.2	3.4	3.1	3.2	5.3	4	1.9	21.4	10.4	7.3	28.3	10.6	8.8
Medicilândia –	LZATM248	M	17.2	9	7.6	6.4	5.6	1.6	1.4	1.6	2	2.1	0.9	9.7	3.9	3	11.9	4.7	3.5
Medicilândia –	LZATM255	F	35	18.6	18.4	13.3	12.8	3.7	2.9	2.7	5	3.9	1.7	21.4	9.3	6.8	28.3	10.4	8.3
Uruará – Brazil	LZATM355	F	41	21.4	19.8	16.1	16.2	3.6	4	3	5.6	5	2.4	22.9	12	7.1	30.7	11.3	11.4
Uruará –	LZATM356	M	25.8	13.3	12.6	9.9	9.6	2.4	2.1	2	3.5	3.8	1.2	16.1	7.1	4.9	21	7.9	6.3
Uruará –	LZATM357	F	25.9	13.2	12.8	10.8	9.8	2.3	2.2	2.4	3.5	3.5	1.3	15.2	7	5.1	20.3	7.5	6.3
Uruará –	LZATM358	F	34	17.9	16.2	12.5	12.2	3	3.5	2.8	4.8	4	1.7	20	9	6.1	25.9	9.8	8.2
Uruará –	LZATM359	F	29.8	16.4	16	12.5	11.5	2.4	2.5	2.4	4.1	4	2.3	17.9	8.8	5.5	23.7	8.8	7.7
Uruará –	LZATM360	F	36.9	18.4	19.5	15	13.9	3.3	3.8	2.9	5.5	4.2	1.7	21.9	9.9	7	30.4	11	8.8
Senador José Porfírio	LZATM 753	F	23.2	11.5	10.7	8.9	7.8	2.1	2	2.1	3.2	2.9	1.2	14.2	5.6	4.6	17.5	7.1	5
Flona Tapajós – Brazil	CTGANSISTA_D_1168	F	41.5	21.7	20.1	14.1	14.7	3.4	3.3	3.3	5.0	4.2	2.0	23.4	10.2	7.5	31.0	10.7	9.3
Flona Tapajós – Brazil	CTGANSISTA_D_1246	F	38.9	21.0	20.7	14.6	13.9	3.0	4.1	3.4	5.3	4.8	2.1	23.9	10.7	7.9	31.6	10.4	9.3
Flona Tapajós –	CTGANSISTA_D_1235	M	39.5	19.3	20.0	14.6	13.6	4.0	4.0	3.6	5.5	5.0	2.3	22.9	10.4	7.4	30.1	11.7	8.2
Flona Tapajós –	CTGANSISTA_D_1145	F	36.6	20.9	19.3	13.8	13.8	2.7	3.7	3.3	5.2	4.3	2.0	22.3	10.5	7.3	29.5	10.8	9.4
Flona Tapajós –	CTGANSISTA_D_1253	F	36.9	20.0	19.3	14.4	13.4	3.1	3.7	3.2	5.6	4.6	2.6	22.0	10.3	7.6	29.2	10.3	8.6
Flona Tapajós –	CTGANSISTA_D_1239	F	39.4	20.7	18.8	14.2	13.8	3.8	4.6	3.2	5.0	4.7	2.1	23.2	10.2	7.6	29.0	11.9	8.2
Flona Tapajós –	CTGANSISTA_D_1275	F	35.5	19.3	18.6	13.6	12.7	3.6	3.6	2.9	4.9	4.5	2.1	21.9	10.1	6.8	28.9	11.4	7.8
Flona Tapajós –	CTGANSISTA_D_1259	M	27.9	14.2	14.2	10.1	9.5	2.6	2.6	2.1	4.0	3.4	1.5	15.9	7.5	5.0	21.0	7.4	6.6
Flona Tapajós –	CTGANSISTA_D_1260	F	28.7	15.0	15.0	10.7	9.7	2.6	2.6	2.3	3.9	4.2	1.7	16.8	7.9	5.7	22.2	8.1	6.3
Flona Tapajós –	CTGANSISTA_D_1257	F	26.4	13.7	13.1	9.4	9.3	2.3	2.5	2.3	3.3	3.7	1.6	15.3	7.4	4.9	20.0	7.6	6.1
Flona Tapajós –	CTGANSISTA_D_1256	M	27.6	15.3	14.6	10.7	10.6	2.6	2.7	2.4	4.1	3.8	1.7	16.9	8.0	5.6	22.0	8.2	6.3
Flona Tapajós –	CTGANSISTA_D_1244	F	31.3	15.5	15.9	12.3	11.3	2.6	3.5	2.7	4.4	4.2	1.9	19.1	8.2	6.0	25.2	9.6	8.1
Flona Tapajós –	CTGANSISTA_D_1240	M	27.7	14.2	14.5	10.7	9.4	2.8	2.5	2.3	3.7	3.5	1.6	16.0	7.6	5.5	22.2	8.0	5.8
Borba – Brazil	INPA-H 34571	M	32.8	16.4	17	12.8	12.3	2.3	3.9	2.5	4.3	4.3	1.8	17.8	9.3	6.5	24.6	8.5	7.7
Borba –	INPA-H 34577	M	30.8	15.3	14.5	11.3	10.7	2.4	3.6	2.9	3.8	4.5	1.5	16.8	7.6	5.6	22.1	8.6	6.4
Borba –	INPA-H 34562	F	32.4	15.7	15.4	11.9	11.1	2.6	4.1	2.7	3.9	4.4	1.8	16.2	7.8	6.5	21.6	7.2	6.7
Borba –	INPA-H 34573	M	34.3	19.1	17.8	12.6	11.6	2.6	4	2.9	4.5	3.9	1.6	19	10.2	6.5	26.2	9	7.7
Borba –	INPA-H 34578	M	28.6	15.4	15.9	10.4	10	2.3	3.5	2.5	3.8	3.4	1.3	17	9.1	5.6	23.8	8.8	6.1
Borba –	INPA-H 34575	M	31.6	15.9	16.3	11.1	10.5	2.3	3.3	2.6	4.2	4	1.4	17.5	8.7	5.7	23.3	8.2	6.8
Borba –	MPEG 7088	F	36	18.7	17.6	14	13.4	3.9	3.5	3.1	5.2	4.2	2.2	20.2	10	7.3	28.3	11.1	9.2
Borba –	INPA-H 34580	M	31.1	14.4	14	11.6	10.2	2.6	3.8	2.8	4.2	4.3	1.6	16	7.4	6	21.1	8	5.7
Borba –	INPA-H 34579	M	30.7	15.3	14.6	11.7	10.8	2.6	3.9	2.9	4	3.8	1.7	16	7.8	5.8	21.9	8.8	6.5
Borba –	INPA-H 34565	M	31.4	15.1	14.1	11.9	10.3	2.8	3.6	3	4.4	4.4	1.7	16.7	8	6.1	21.6	8.5	6.2
