# Corrigenda: A new species of *Pristimantis* from eastern Brazilian Amazonia (Anura, Craugastoridae). ZooKeys 687: 101–129 (2017). https://doi.org/10.3897/zookeys.687.13221

**DOI:** 10.3897/zookeys.711.21220

**Published:** 2017-10-23

**Authors:** Elciomar Araújo De Oliveira, Luís Reginaldo Ribeiro Rodrigues, Igor Luis Kaefer, Karll Cavalcante Pinto, Emil José Hernández-Ruz

**Affiliations:** 1 Programa de Pós-Graduação em Biodiversidade e Biotecnologia da Rede BIONORTE, Universidade Federal do Amazonas, Av. Gen. Rodrigo Octávio Jordão Ramos, 3000, CEP 69077-000, Manaus, Amazonas, Brazil; 2 Docente do PPG Bionorte, Universidade Federal do Oeste do Pará; 3 Universidade Federal do Amazonas. Av. Gen. Rodrigo Octávio Jordão Ramos, 3000, CEP 69077-000, Manaus, Amazonas, Brazil; 4 Biota Projetos e Consultoria Ambiental LTDA, Rua 86-C, 64, CEP 74083-360, Setor Sul, Goiânia, Goiás, Brazil; 5 Programa de Pós-Graduação em Biodiversidade e Conservação, Faculdade de Ciências Biológicas, Campus Universitário de Altamira, Universidade Federal do Pará, Rua Coronel José Porfírio, 2515, CEP 68372-040, Altamira, Pará, Brazil

In our recent work on the description of the new species of *Pristimantis* (De Oliveira et al. 2017), some errors that can impair your complete understanding were not noticed in the text. Corrections are as follows:

**Material and methods** (pag. 102)

In the section **Morphological analyzes** where it is written “twenty-two belonging to type series” read “twenty-nine belonging to type series”.

**Table 1** (p. 106)

Line 11 should be removed from the **Table 1** because this sequence was not used.

Individuals “BIOTA 1218, BIOTA 1111, MPEG 095, MPEG 109, MPEG 160, MPEG 165 e MPEG 177” are not part of the type series.

**Table 1** (p. 106 and 107)

In the **Table 1**, individuals belonging to the Borba 1 group are INPA-H 34565, 34579 and 34580, while those belonging to the Borba 2 group are INPA-H 34571, 34577, 34562, 34573, 34578 and 34575.

**Bioacoustic analysis** (p. 111)

In **Figure 3C**, the vocalization belongs to populations from Bolivia (Padial and De La Riva 2009). The oscillograms were retrieved from Padial and De La Riva (2009). José M. Padial is the author of figure 3B, Ignacio De La Riva is the author of figure 3C and J. Kohler is the author of figure 3D.

**Paratopotypes** (p. 113)

Where it is written “and nine adult females:” read “and eight adult females:”. Individual “LZATM 742” is mentioned twice, but it consists of only one.

**Diagnosis** (p. 114)

Where it is written “*dorsolateral fold absent*” read “*dorsolateral fold present*”.

**Table 4** (p. 115)

Add “Obs: The (*) indicates description by Duellman and Lehr (2009) for *Pristimantis fenestratus* Peru and (**) indicates description by Padial and De La Riva (2009) for *Pristimantis fenestratus* Bolivia”.

**Appendix 2** (pag. 129)

In the column **locality**, individuals from “Borba” should not be part of this table, since they do not belong to the taxon *Pristimantis latro*.
